# Detection of Aflatoxin B_1_ Based on a Porous Anodized Aluminum Membrane Combined with Surface-Enhanced Raman Scattering Spectroscopy

**DOI:** 10.3390/nano10051000

**Published:** 2020-05-24

**Authors:** Yanting Feng, Lei He, Ling Wang, Rijian Mo, Chunxia Zhou, Pengzhi Hong, Chengyong Li

**Affiliations:** 1 College of Food Science and Technology, Guangdong Ocean University, Zhanjiang 524088, China; yanting_feng@163.com (Y.F.); wl6509@163.com (L.W.); chunxia.zhou@163.com (C.Z.); hongpengzhi@126.com (P.H.); 2 Shenzhen Institute of Guangdong Ocean University, Shenzhen 518108, China; airyhelei@163.com; 3 School of Chemistry and Environment, Guangdong Ocean University, Zhanjiang 524088, China; 4 Southern Marine Science and Engineering Guangdong Laboratory, Zhanjiang 524088, China

**Keywords:** surface-enhanced Raman scattering (SERS), Ag nanoparticles, porous anodized aluminum membrane, Aflatoxin B_1_

## Abstract

An Aflatoxin B_1_ (AFB_1_) biosensor was fabricated via an Ag nanoparticles assembly on the surface of a porous anodized aluminum (PAA) membrane. First, the Raman reporter 4-Aminothiophenol (4-ATP) and DNA (partially complementary to AFB_1_ aptamer) were attached to the surface of Ag nanoparticles (AgNPs) by chemical bonding to form a 4-ATP-AgNPs-DNA complex. Similarly, the surface of a PAA membrane was functionalized with an AFB_1_ aptamer. Then, the PAA surface was functionalized with 4-ATP-AgNPs-DNA through base complementary pairing to form AgNPs-PAA sensor with a strong Raman signal. When AFB_1_ was added, AgNPs would be detached from the PAA surface because of the specific binding between AFB_1_ and the aptamer, resulting in a reduction in Raman signals. The detection limit of the proposed biosensor is 0.009 ng/mL in actual walnut and the linear range is 0.01–10 ng/mL. The sensor has good selectivity and repeatability; it can be applied to the rapid qualitative and quantitative detection of AFB_1_.

## 1. Introduction

Aflatoxins are listed as Class I carcinogen by the World Health Organization [1]. Among them, Aflatoxin B_1_ (AFB_1_) has increasingly attracted public attention because of its strongest toxic effect and high carcinogenicity [2,3,4]. Aflatoxin is widely used in food ingredients such as peanuts, milk, cereals [5,6]. It is not easily inactivated during cooking. Due to the serious harm to humans and animals, many countries have strict regulations on the limits of aflatoxins B_1_ in foods [7,8]. For example, the limit in Romania and European Commission (EU) is 2 ng/mL, and in Australia is 5 ng/mL.

There are many traditional techniques that have been developed to detect AFB_1_ in multifarious samples, including gas chromatography (GC) [9], high-performance liquid chromatography (HPLC) [10], enzyme-linked immunosorbent assay (ELISA) [11,12], and LC coupled with tandem mass spectrometry (LC–MS/MS) [13]. However, these methods usually need precise equipment, professional technical personnel, and generally require hours or days to obtain data. Therefore, it is necessary to develop a reliable, convenient method for detecting AFB_1_

Surface-enhanced Raman scattering (SERS) has been applied in many fields [14], such as food chemistry [15,16,17], environmental science [18], and biotechnology [19,20,21]. At present, there are many reports about detection of AFB_1_ via SERS technology [22,23,24,25,26]. A popular SERS substrate consists of noble metallic nanostructures, which can sustain surface plasmon resonance and enhancing the signal of Raman reporter [27,28,29]. Nanorods, nanostars [30] and nanoparticles [31] are often used as substrates. Among them, Ag nanoparticles (AgNPs) are a common substrate due to its simple preparation and higher activity than gold and copper [26]. Li et al. [29]. It has been reported that the gold nanostar core–Ag nanoparticle satellite sensor can be used to detect AFB_1_ content in peanut milk. The regression coefficient was 0.995 and the limit of detection (LOD) was 0.48 pg/mL. However, this method will cause the dispersed nanoparticles easily agglomerate in solution. As a typical example of a biosensor, a porous anodized aluminum (PAA) membrane has the characteristic features of a surface modification, good mechanical stability and easy preparation [32,33,34,35,36]. If a PAA membrane is combined with SERS technology, it can prevent the aggregation of free nanoparticles in solution and also reduce the relative standard deviation [16,20]. Liu et al. [20] showed that vancomycin (Van)-coated Ag nanoparticles were used as a SERS substrate, which can be used for label-free bacterial analysis on a PAA nanochannel array. However, this method can only provide qualitative analysis of the sample.

**Scheme 1:**
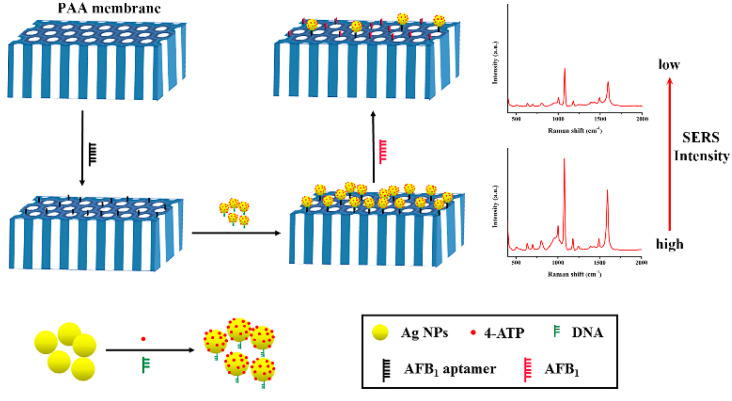
Schematic illustration of the fabrication of Surface-enhanced Raman scattering (SERS) active Ag nanoparticles-porous anodized aluminum (AgNPs-PAA) sensor assemblies for the detection of Aflatoxin B_1_ (AFB_1_).

In this work, a new type of AgNPs-PAA biosensor was fabricated to detect AFB_1_ based on aptamer competition recognition, as shown in Scheme 1. First, 4-ATP-AgNPs-DNA complexes were prepared by modifying DNA (partially complementary to the AFB_1_ aptamer) and 4-aminothiophenol (4-ATP, the Raman reporter) on the surface of AgNPs. At the same time, the surface of the PAA membrane was functionalized with AFB_1_ aptamer. Then, the PAA membrane surface was functionalized with 4-ATP-AgNPs-DNA by the base-pairing of aptamer and DNA. In the presence of AFB_1_, it will compete with DNA for aptamers, resulting in the detachment of ATP-AgNPs-DNA from a PAA membrane and a reduction in 4-ATP Raman intensity. This method can achieve high sensitivity detection of AFB_1_ in food.

## 2. Materials and Methods

### 2.1. Reagents and Materials

Silver nitrate (AgNO_3_) was bought from Shanghai Aladdin Biochemical Technology Co., Ltd. (Shanghai, China). Sodium citrate (Na_3_C_6_H_5_O_7_·_2_H_2_O) was supplied by Shanghai Macklin Biochemical Co., Ltd. (Shanghai, China). (3-Aminopropyl) trimethoxysilane (APTMS) and 4-Aminothiophenol (4-ATP, C_6_H_7_NS) were bought from Adamas Reagent, Ltd., (Shanghai, China). AFB_1_ aptamer (NH_2_-5′-GTTGGGCACGTGTTGTCTCTCTGTGTCTCGTGCCCTTCGCTAGGCCCACA-3′) and SH-DNA (HS-5′-CAGAGAGACAACACGTGCCCAAC-3′, partially complementary of AFB_1_ aptamer) were bought from Sangon Biotech Co., Ltd. (Shanghai, China). Aluminum foil with a thickness of 0.1 mm and a purity of 99.999% was from Trillion Metals Co., Ltd. (Beijing, China). All other reagents were analytically pure and used without purification. All of the solutions were prepared with ultrapure water (Milli-Q, Merck, Darmstadt, Germany).

### 2.2. Instrument

UV-Visible absorption spectra were obtained with a (Hitachi, U-3900H) spectrometer (Beijing, China). Transmission electron microscopy (TEM, JEOL, JEM-1400, Akishima City, Tokyo, Japan) was performed at 80 kV to determine the sizes of AgNPs.

Scanning electron microscopy (SEM) was performed at 5 kV to observe the shape of PAA (Tescan Co., Ltd., TMIRA3, Czech Republic). Portable Raman spectrometer (Ocean Optics, SR-510 Pro, Beijing, China) was used to acquire the spectral signals.

### 2.3. Preparation of 4-ATP-AgNPs-DNA

At first, 50 nm AgNPs were prepared via reducing sodium nitrate solution with sodium citrate. 2 mL of 1% sodium citrate solution was quickly added to the boiling silver nitrate solution (100 mL, 0.18 g/L) and reacted for 1 h. The final color of the solution was yellow-green, and it was stored at 4 °C after the silver colloid was cooled down. One mL AgNPs were centrifuged (8000 rpm, 10 min) and re-dispersed in 900 µL 10 mM PBS (pH 7.4) buffer. Then, 10 µL 10 μM SH-DNA (complementary to aptamer), 10 µL 10 μM 4-ATP and 100 µL 100 mM sodium chloride solution was added sequentially. Next, it was incubated at 37 °C for 8 h to form the 4-ATP-AgNPs-DNA complex. After the reaction, the excess DNA was removed by centrifugation (8000 rpm, 10 min) and washed 3 times. Finally, the precipitate was suspended in 1 mL 10 mM PBS buffer.

### 2.4. Preparation and Surface Modification of PAA Membrane

As previously reported, a pore diameter of 20 nm PAA membrane was prepared by a two-step anodization method [37]. Briefly, high-purity (99.999%) aluminum foils were annealed at 500 °C for 2 h. Then the aluminum was placed in acetone for 5 min. The aluminum foils were put into the mixture of perchloric acid/ethanol (1:4, *v/v*) for electrochemical polishing (20 V, 3 min) before the anodization. The first anodization was carried out under a constant cell potential of 27 V in 0.3 M sulfuric acid solution at 2 °C for 2 h. The first anodization aluminum was foiled in a mixture of 6 wt% phosphoric acid and 1.8 wt% chromic acid at 60 °C for 40 min. The conditions of second anodization were the same as above, and the oxidation time was 10 h. After the anodization was completed, the aluminum foils put in to the mixture of perchloric acid/ethanol (1:1, *v/v*) for electrochemical peeling (37 V, 3 s).

The PAA membrane was boiled in 30% H_2_O_2_ for 15 min. Then, PAA membrane was soaked in 5% APTMS (acetone solution) for 6 h to surface silanization, rinsed thoroughly with acetone and water, followed by drying at 110 °C for 1 h to form a silane layer. Next, it was soaked in 2.5% glutaraldehyde at 4 °C for overnight. PAA membrane was immersed at 20 µM AFB_1_ aptamer for 12 h at 4 °C. Finally, the remained aldehyde groups on the PAA surface were terminated with 0.2% n-propylamine, and then the PAA was washed with NaCl (0.1 M) solution and ultrapure water following this order.

### 2.5. Fabrication of AgNPs-PAA Biosensor

The AgNPs-PAA sensor was fabricated as follows. Briefly, PAA membrane was immersed in 300 µL of 4-ATP-AgNPs-DNA in the dark for 2 h, then washed with water. During this process, the color of the PAA membrane changed from white to gray. Then tape was used to connect the side without AgNPs to the aluminum foil for easy detection.

### 2.6. Detection of AFB_1_ with AgNPs-PAA Biosensor

To investigate whether the AgNPs-PAA sensor can be used for AFB_1_ detection, the AgNPs-PAA was immersed in 300 μL AFB_1_ standard solution (methanol as a solvent) of different concentrations (0, 0.1, 0.5, 1, 5, 10 ng/mL) in the dark for 2 h, then washed with water and dried at room temperature. Finally, the Raman signal of the sensor was detected by the Raman spectrum. The Raman probe was used to detect the PAA membrane in a card slot (Ocean Optics, Beijing, China). The probe working distance was 7.5 mm and the spot diameter was less than 2 mm. The excitation wavelength of the Raman spectrum was 785 nm. Without special introduction, the measured Raman intensity was from three different points on the PAA membrane, and the integration time was 3 s.

At the same time, the AgNPs-PAA biosensor was also used to detect AFB_1_ in walnuts. Before detecting, walnut samples need to be pre-treated as follows: 1.00 g of walnuts sample was ground and dried thoroughly. Two mL of methanol/water (volume ratio of 8:2) was added. Then, it was sonicated for 0.5 h and centrifuged for 20 min (12,000 rpm). One mL extraction solution was collected and AFB_1_ was added to form different concentration of AFB_1_ (0, 0.01, 0.05, 0.1, 1, 5, and 10 ng/mL). The following steps were consistent with the detection of the AFB_1_ described above.

### 2.7. Detection of Specificity and Repeatability in AgNPs-PAA Biosensor

To investigate the specificity of the prepared biosensor, three mycotoxins (AFG_1_, AFB_2_ and OTA) were used as negative control 10 ng/mL of AFG_1_, AFB_2_, OTA and AFB_1_ standard were added to walnut samples to prepare corresponding sample solutions. The following steps were consistent with the detection of the AFB_1_.

To study the repeatability of the sensor, the same batch of 30 AgNPs-PAA biosensors were soaked in 0.1 ng/mL AFB_1_ spiked walnut sample for 2 h in the dark, then washed with water and dried at room temperature. Finally, the Raman signals of the sensor were detected. The detection process was the same as the detection AFB_1_ described above.

## 3. Results and Discussion

### 3.1. Representation of Sensors

**Figure 1:**
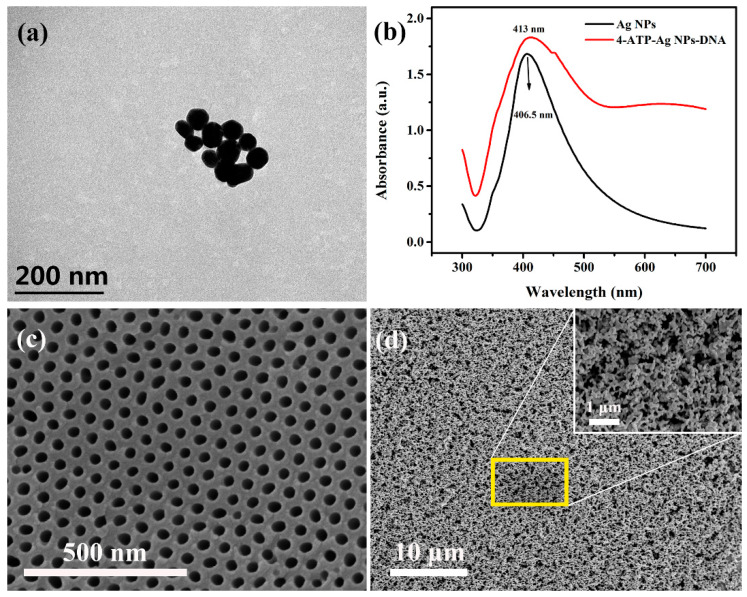
The characteristics of the biosensor. (**a**) Transmission electron microscopy (TEM) image of AgNPs. (**b**) Ultraviolet-visible (UV-Vis) absorption spectra of AgNPs and 4-Aminothiophenol (4-ATP)-AgNPs-DNA. (**c**) Scanning electron microscopy (SEM) image of top surface of PAA membrane. (**d**) SEM image of PAA membrane functionalized with AgNPs (Inset: magnified view).

The TEM image of AgNPs as shown in Figure 1a, the average size of the AgNPs ranged from 50 to 60 nm. As shown in Figure 1b, the UV-vis absorption peak width of AgNPs is narrow, indicating the particle size of AgNPs is average. The localized surface plasmon resonance (LSPR) peak of the AgNPs is 406.5 nm. The spectrum of 4-ATP-AgNPs-DNA shows a red shift of 6.5 nm. This indicates that DNA and 4-NTP are attached on the surface of AgNPs and changed the constant around the dielectric AgNPs. On the other hand, a broad band appeared at 625 nm can be prove that the distance between the AgNPs has changed and some small aggregates have formed after surface modification. Figure 1c shows that the average pore diameter of the PAA membrane is about 20 nm, and the arrangement is regular. The surface of the membrane is clean, which facilitates functional modification. Figure 1d shows the SEM image of PAA membrane functionalized with AgNPs. A large number of AgNPs based pairing of aptamer and DNA, are captured on the surface of the PAA membrane to form a uniform Ag layer. It means that the 4-ATP-AgNPs-DNA biosensor has been fabricated successfully.

### 3.2. Selection of Substrate in SERS Sensor

**Figure 2:**
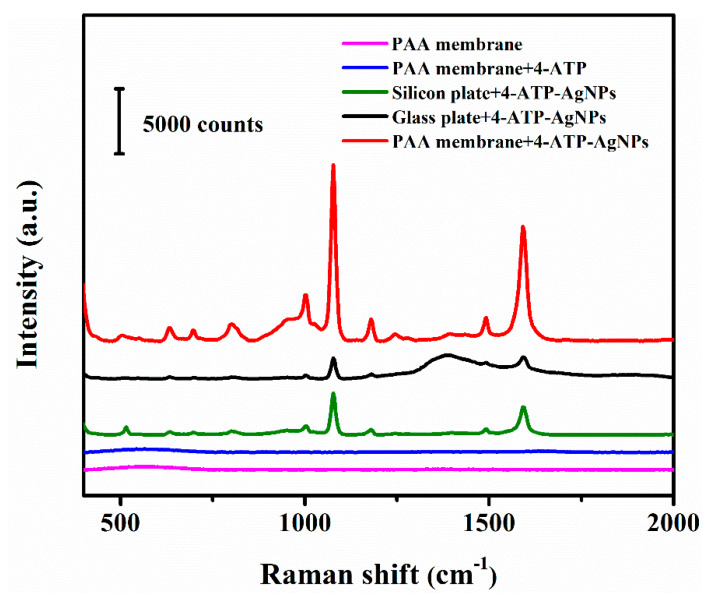
Raman spectroscopy measurement on different substrates.

The Raman signal of 4-ATP is difficult to detect in aqueous solution [29], so the Raman detection took place after drying. The Raman signal of 4-ATP solution with and without AgNPs is presented in Figure 2. Compared with no added AgNPs (Figure 2, blue curve), the addition of AgNPs can significantly enhance the characteristic peak of Raman reporter 4-ATP and the Raman intensity of 4-ATP has a maximum value at 1080 cm^−1^ (Figure 2, red curve). Therefore, in the subsequent Raman analysis, AgNPs are used to enhance the signal, and the peak intensity at 1080 cm^-1^ was used for SERS intensity analysis.

In order to explore whether the PAA membrane is a suitable Raman substrate, it is compared with other Raman substrates, such as glass sheets and silicon wafers, as shown in Figure 2. When the glass sheet was used as a Raman substrate, a broad peak appears at 1400 cm^−1^ [38] and the characteristic peak of 4-ATP (1080 cm^−1^) is small (Figure 2, black curve). Compared with the Raman signal using a silicon wafer as a substrate (Figure 2, green curve), the Raman signal of 4-ATP with the PAA membrane as the substrate is stronger (Figure 2, red curve). The reason is that the rough surface of PAA promotes Raman enhancement effect in some extent [39]. In addition, the PAA membrane does not affect the signal of surface plasmon enhancement (Figure 2, pink curve). Therefore, the PAA membrane is selected as the Raman substrate.

### 3.3. SERS Detection of AFB_1_ in Standard Solution and Walnut Sample

**Figure 3:**
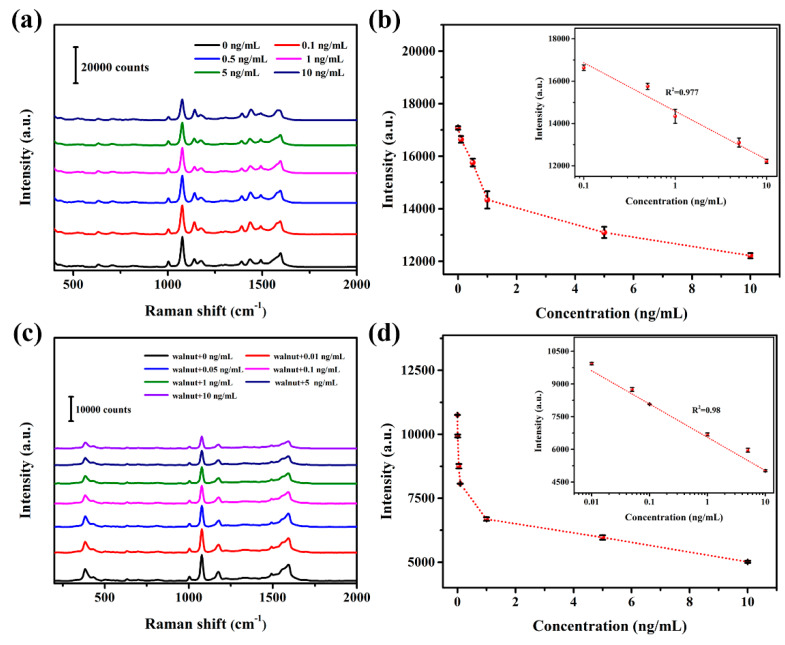
Surface-enhanced Raman scattering (SERS) spectra (**a**) and standard curve (**b**) for detecting different concentrations of AFB_1_ standard solution by AgNPs-PAA biosensor (Inset: the linear fitting of AFB_1_ from 0.1 ng/mL to 10 ng/mL). A SERS spectra (**c**) of walnut spiked sample and (**d**) standard curve (Inset: the linear fitting of AFB_1_ from 0.01 ng/mL to 10 ng/mL).

Figure 3a shows the Raman spectra of AFB_1_ at different concentrations detected by the AgNPs-PAA sensor. The SERS intensity of 4-ATP decreases with the increase of AFB_1_ concentration in the range of 0.1 to 10 ng/mL. This is because AFB_1_ preferentially binds to aptamers, causing 4-ATP-AgNPs-DNA to detach from the surface of PAA. Therefore, the Raman signal is decreased. Figure 3b is a linear plot of Raman signal intensity at 1080 cm^−1^ versus AFB_1_ concentration, which is linearly correlated in the range of 0.1 to 10 ng/mL. The linear regression equation is *y* = 14,582.673 − 2285.185lg*x*, the correlation coefficient R^2^ = 0.977, and the limit of detection of AFB_1_ is 0.083 ng/mL (LOD = 3SD/k, where SD and k are the standard deviation of the blank and the slope of the calibration graph respectively).

The sensor’s ability to detect interstitial samples has been verified through the addition of AFB_1_ in walnut samples. When the walnut samples were tested, it was found that the concentration is not within the linear range of AFB_1_ standard solution mentioned above (Figure 3a,b). This phenomenon may be due to the presence of some complex compounds (such as fatty acids, carbohydrates [40,41]) in the walnut samples that interfere with the Raman signal. Therefore, the calibration curve of the standard sample is not applicable to the actual sample, and a new standard curve will be required when the actual sample is tested. Here, AFB_1_ standard solutions of different concentrations (0, 0.01, 0.05, 0.1, 1, 5, 10 ng/mL) were added to walnut samples to create a new standard curve and calculate the concentration of AFB_1_ in walnuts, as demonstrated in Figure 3c,d. The Raman signal decreases with increasing AFB_1_ concentration (Figure 3c). Figure 3d shows the linear relationship between Raman intensity of 4-ATP and AFB_1_ concentration at 1180 cm^−1^. The concentration range of AFB_1_ is 0.01 to 10 ng/mL. The linear regression equation is *y* = 6555.400 − 1528.247lg*x*, the correlation coefficient R^2^ = 0.980, and the limit of detection is 0.009 ng/mL. After calculation, AFB_1_ in walnut is approximately 0.002 ng/mL. It can be considered that the concentration of AFB_1_ in walnut sample is negligible, because the calculation result is lower than the minimum detection limit of the sample. However, the method is still suitable for the detection of actual samples, because the EU limit (2 ng/mL) for AFB_1_ in food is within the linear range of the method.

### 3.4. Specificity and Repeatability of AgNPs-PAA Biosensor

**Figure 4:**
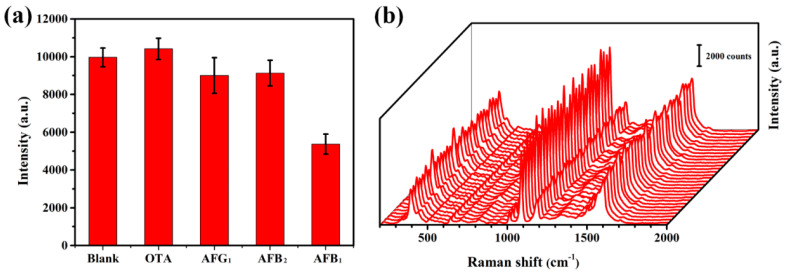
Specificity and repeatability of AgNPs-PAA sensor. (**a**) SERS intensity of the sensor to detect AFB_1_, AFB_2_, AFG_1_, OTA. The concentration of samples was 10 ng/mL. Error bars show the standard deviation of three experiments; (**b**) SERS spectra of 0.1 ng/mL AFB_1_ spiked samples, repeated 30 times.

As shown in Figure 4a, the concentrations of all mycotoxins are 10 ng/mL, and the test results are the similar level as the blank samples. In contrast, the intensity of SERS obtained by testing AFB_1_ is lower than that of the negative control. The results confirm that the AgNPs-PAA sensor has an excellent selectivity to detect AFB_1_ based on the specific recognition of the AFB_1_ aptamer.

Signal instability is a common situation in Raman detection, which is caused by the uneven distribution of nanoparticles during the fabrication process of SERS substrates. The detection efficiency of AgNPs-PAA sensor is affected from reproducibility. The reproducibility of this method was studied. The statistical Raman spectrum is shown in Figure 4b. The relative standard deviation (RSD) of the Raman peak at 1180 cm^−1^ is 11.14%. This indicates that the AgNPs-PAA sensor has good reproducibility in actual sample detection.

## 4. Conclusions

In this study, an AgNPs-PAA biosensor is constructed based on aptamer competition recognition to achieve rapid detection of AFB_1_. The results show that Raman intensity decreases as the AFB_1_ concentration increasing. There is a good linear relationship between the range 0.01–10 ng/mL (R^2^ = 0.980) and the detection limit is approximately 0.009 ng/mL in actual samples. The sensor has good selectivity and repeatability and it can be applied to the rapid qualitative and quantitative detection of AFB_1_.
